# Ethnomedicinal and cultural practices of mammals and birds in the vicinity of river Chenab, Punjab-Pakistan

**DOI:** 10.1186/s13002-017-0168-5

**Published:** 2017-07-12

**Authors:** Muhammad Altaf, Arshad Javid, Muhammad Umair, Khalid Javed Iqbal, Zahid Rasheed, Arshad Mehmood Abbasi

**Affiliations:** 1 Department of Zoology, Women University of Azad Jammu and Kashmir, Bagh, Pakistan; 2 Department of Wildlife and Ecology, University of the Veterinary and Animal Sciences, Lahore, Pakistan; 3 School of Agriculture and Biology, Shanghai Jiao Tong University, Shanghai, China; 4 Department of Life Sciences, Islamia University Bahawalpur, Bahawalpur, Pakistan; 5 Department of Mathematics, Women University of Azad Jammu and Kashmir, Bagh, Pakistan; 6 Department of Environment Sciences, COMSATS Institute of Information Technology, Abbottabad, Pakistan

**Keywords:** Ethnomedicinal uses, Mammals, Birds, Cultural significance, Pakistan

## Abstract

**Background:**

Although, use of animal species in disease treatment and culture practices is as ancient as that of plant species; however ethnomedicinal uses and cultural values of animal species have rarely been reported. Present study is the first report on the medicinal uses of mammals and bird species in Pakistan.

**Methods:**

Questionnaires and semi-structured interviews were applied to collect qualitative and quantitative data from local informants (*N* = 109). Relative frequency of mention (RFM), fidelity level (FL), relative popularity level (RPL), similarity index (SI) and rank order priority (ROP) indices were used to analyzed the data.

**Results:**

One hundred and eight species of animals, which include: 83% birds and 17% mammals were documented. In total 30 mammalian and 28 birds’ species were used to treat various diseases such as rheumatic disorders, skin infections and sexual weakness among several others. Fats, flesh, blood, milk and eggs were the most commonly utilized body parts. *Bos taurus, Bubalus bubalis, Capra aegagrus hircus, Felis domesticus, Lepus nigricollis dayanus* and *Ovis aries* (mammals) and *Anas platyrhynchos domesticus, Columba livia, Coturnix coturnix, Gallus gallus* and *Passer domesticus* (birds) were the highly utilized species. Medicinal and cultural uses of 30% mammals and 46% birds were reported for the first time, whereas 33% mammals and 79% birds depicted zero similarity with previous reports.

**Conclusion:**

Present study exhibits significant ethnozoological knowledge of local inhabitants and their strong association with animal species, which could be helpful in sustainable use of biodiversity of the region. Additionally, in vitro and in vivo evaluation of biological activities in the mammalian and birds’ species with maximum fidelity level and frequency of mention could be important to discover animal based novel drugs.

**Graphical Abstract:**

Some commonly used mammals and birds species of the study area
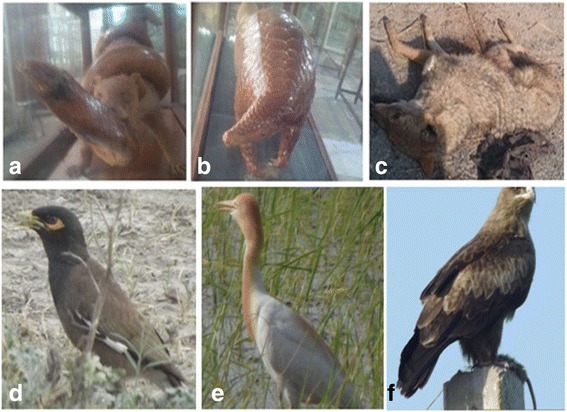

## Background

Animal resources have been of significant value in different features of human life from its origins. Various animal species are present in art, music, religion, literature, medicine, food and many other human expressions [1, 2]. The phenomenon of zootherapy is noticeable mutually by a broad geographical distribution and profound historical origins [3]. Zootherapy contributes significantly in the healing practices, magic rituals [4] and constitutes an important alternative in modern civilization [5]. Therefore, to recognize this important relationship, ethnozoology should be considered as an affective field [6], and the social and cultural bonds between native people and animal species should be taken into account [7]. The use of animals for medicinal purposes is part of a body of traditional knowledge [5]. Wild and domestic animals and products derived from their bodies are not only used in traditional medicines, but are also increasingly valued as raw materials in the preparation of modern medicines and herbal preparations [8], 8.7% of essential chemicals are derived from animals [9]. Regardless of their importance, studies on the therapeutic uses of animals and their body parts have been neglected, when compared to plants [5].

Rural people make use of a large host of existing resources; while, they are not all evenly important. The idea of cultural importance arose through the study of traditional systems of classification and taxonomy [10]. Cultural importance of a species is the value of its characteristic within a human ethnic group [11]. There are different selection parameters of specific species or groups of species [12–14]. The idea of a species, its specific ecological characteristics, the benefits obtained from it, the direct and/or indirect harm or damage it can cause, it’s cultural importance, and other criterion, are illustrations of substantial and insubstantial characteristics that people take into consideration to allocate value [15, 16]. And such evaluation involves different ecological and social procedures which are specific to each human ethnic group and occur in a different way through era. Thus, the cultural importance of an animal is a scientific method [17].

The fundamental relation between humans and animals goes behind utilitarian features. Consequently, documentation of traditional knowledge associated with medicinal and cultural uses of the wild and domesticated animal species is essential because the majority of local communities are rapidly losing their socioeconomic and cultural characteristics [18]. Particularly, mammals and birds are known as the most important and extremely fascinating species that is present in people’s thoughts and cultural traditions [16]. In several human ethnic communities, mammals and birds species constitute the major source of protein; used in medicine, leather industry as well as in folklore [16, 19–21]. Pakistan has a rich diversity of mammals with a total of 195 listed species [22], and birds with a total of 668 observed species [23] and majority of them are utilized in traditional health care. However, ethnomedicinal uses and cultural importance of mammals and birds species in Pakistan have never been documented. Present study was aimed to document the medicinal uses and cultural value of mammals and birds species used by the local communities of three districts: Sialkot, Gujrat and Gujranwala around the river Chenab in the Punjab province of Pakistan.

## Methods

### Study area

**Fig. 1:**
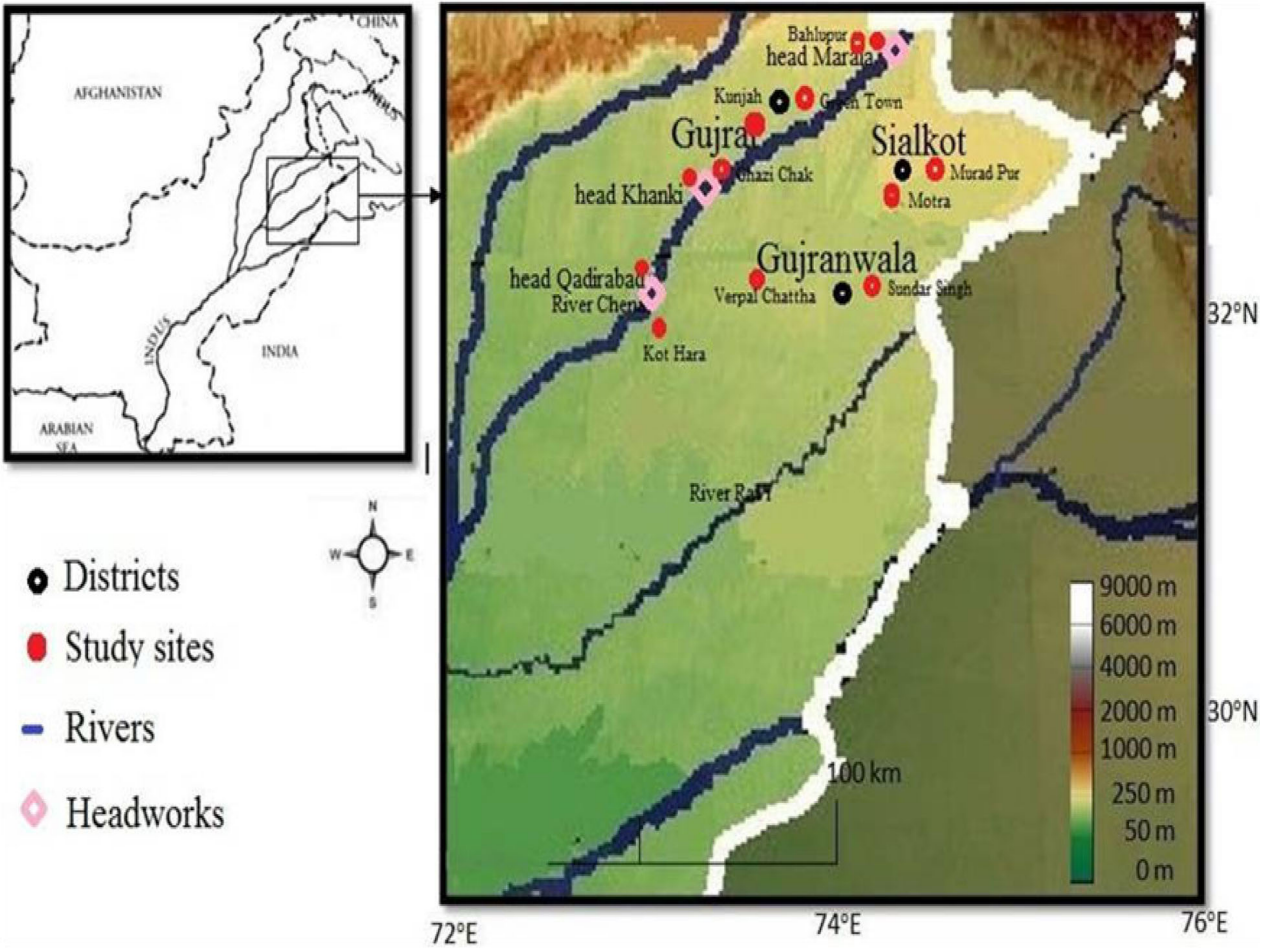
Map showing study area along with visited sites

Present study was conducted in the three districts of Punjab province Pakistan viz. Sialkot, Gujrat and Gujranwala located around the river Chenab (Fig 1). The river Chenab originates from Kangra and Kulu districts of Himachal Pradesh India and enters in Pakistan near Diawara village of district Sialkot [24]. The study area spreads over 9830 Km^2^ with temperature ranges from 1 °C to 48 °C in the months of December and June, respectively [25–28].

### Ethnography

Male population is dominant in the study area, and is estimated around 52%, while remaining 48% are female. Majority of the inhabitants (65%) lives in rural areas and 35% are settled in urban areas. Mughal, Jutt, Arain, Gujjar, Sheikh, Malik, Butt and Rana are the major ethnic communities while Christians are in minority. Most of the inhabitants speak Punjabi language (90.6%), followed by Urdu (9%), Pashto (0.2%), Siraki (0.19%) and English (0.01%). Wheat is the major cereal crop with annual production 1530 Thousand Million Tones (TMT) followed by rice (964 TMT) and sugarcane (225 TMT). Guava and citrus are the major fruits of the area with an annual production of 20,335 and 5010 Million Tons (MT). Study area is well known for vegetable production where potato, onion, tomato, carrot, brinjl, ladyfinger and garlic are commonly grown. Almost 1,347,000 cattle are slaughter annually for meet and on average 211 MT per annum wool obtained from animals like sheep and goats [25–27].

### Data collection and analysis

Field surveys were conducted during 2014–2015 to collect information on ethnomedicinal application of mammals and birds species. Formal consent was received from informants regarding data collection and publication; then the Participatory rural appraisal (PRA) approach as mentioned in the Kyoto Protocol was applied with the consent of the informant. Ethical guidelines of the International Society of Ethnobiology (http://www.ethnobiology.net/) were strictly followed. Questionnaires and semi-structured interviews were conducted from 109 informants (i.e. farmers, teachers, herdsmen, hunters and traditional health practitioners). Informants were selected based on their traditional knowledge on medicinal and cultural importance of mammals and birds species. Mammals species were recognized using field guides “Mammals of Pakistan” [29, 30]. Books of “Birds of Pakistan” were consulted for identification of birds of the study area [31, 32].

Data on ethnomedicinal uses and cultural values were analyzed using various indices such as; relative frequency of mention (RFM), fidelity level (FL), relative popularity level (RPL), rank order priority (ROP) and similarity index (SI).

**Relative frequency of mention (RFM)**: The value of RFM for species of medicinal animals is based on the citing percentage of local informants for that particular animal species. RFM was calculated using formula as reported by [33].

$$ \mathrm{RFM}=\frac{\mathrm{FM}}{\mathrm{N}}\ \left(0\le RFM\le 1\right) $$

Where, FM = Frequency of mention (or number of informants) for a cultural use of a particular species. N = total number of informants.

**Fidelity level (FL):** was calculated by modified formula of [34].

$$ \mathrm{FL}\ \left(\%\right)={\mathrm{N}}_{\mathrm{p}}/\mathrm{FM}\times 100 $$

Where, N_p_ is the number of informants of major ailment (IMA) for particular types of mammals and birds species. FM = Frequency of mention (or number of informants) for cultural use of a particular species.

**Relative popularity level (RPL):** was determined as explained earlier [35, 36]. Briefly, mammals and birds species were divided into two groups ‘popular’ and ‘unpopular’. Popular mammals and birds are those which were mentioned for more than half of the maximum FM. The remaining mammals and birds’ species were noted as unpopular. A co-ordinate system was utilize in which X-axis represents to the FM citing a mammals and birds species for cultural use, while Y-axis represents to the number of different cultural uses for each mammals and birds species. For mammals and birds species with low popularity level, a linear increase was assumed, namely, a greater FM cited the mammals and birds species for any use, hence a greater average number of uses per mammals and birds species. On the other hand, for popular mammals and birds species a horizontal line was supposed namely, the average number of uses per mammal and bird is independent of the FM, who knows the mammalian and avian species; Hence, the average number of uses of a popular mammals and birds species does not increase with the increased FM who mention the mammals and birds species for any medical use. For popular mammalian and avian species, the RPL was selected to 1.0. For mammals and birds species within the unpopular group, the RPL is less than 1.0. RPL values may be noted for each particular mammalian and avian species in accordance with its location on the graph.

**Rank order priority (ROP):** is used to rank the mammals and birds species [35, 36] and was calculated by formula,

$$ \mathrm{ROP}=\mathrm{FL}\times \mathrm{RPL} $$

*Similarity index* (SI) was calculated using formula

$$ \mathrm{SI}={\mathrm{M}}_{\mathrm{s}}/{\mathrm{M}}_t\kern1em \left(0\le \mathrm{SI}\le 1\right) $$

M_s._ = Similar number of medicinal applications in present and previous research records for a particular species.

M_t._ = Total number of medicinal applications in present and previous research records for a particular species.

## Results and discussion

### Demographic features of respondents

A total of 109 informants between the age of 20 to 70 years were interviewed (Table 1). Maximum respondents 73 were 41 to 60 years old. Approximately, 71 were literate with different levels of education viz., primary (25), secondary school certificate (41), graduate (4) and post-graduate (1). About 84 respondents were from rural areas and their main source of income was agriculture. The old age informants possess significant traditional knowledge compared to younger. This may be due to their wide interaction with animal species.

**Table 1:** Ethnographic data of local informants

Variables	Demographic categories	Number of informants
Gender	Male	109
	Female	0
Experience	Health practitioners	20
	Farmer	43
	Teachers	23
	Herdsman	15
	Hunters	9
Age group	20–30	9
	31–40	27
	41–50	36
	51–60	26
	above 60	11
Education	Post-graduate	1
	Graduate	4
	SSC	41
	Primary	25
	Illiterate	38
Residence	Rural	84
	Urban	25
Religious background	Muslim	108
	Non-Muslim	1

### Local nomenclature

Vernacular nomenclature represents the local names of animal species used for medicinal and cultural purposes. Local name usually have clue about habitat, morphological difference, myth and social associations. For example, choha is used as suffix synonym in five species such as *Millardia meltada* (Fasli choha), *Mus musculus* (Chota choha), *Nesokia indica* (Choti push wala choha), *Rattus rattus* (Wada choha) and *Tatera indica* (Jangli choha). These variations in local names are due to difference in morphological characteristics i.e. house rat has larger size and is known as ‘wada choha’; mouse has smaller size and is named ‘chota choha’; and short tailed mole rat is called ‘chhoti dum wala choha’. *Suncus etruscus* (Mediterranean pygmy shrew) is the world smallest mammal. In the study area it is named as ‘choti chachondar’; alike suffix ‘waddi chachondar’ is used for *Suncus murinus* (House shrew) due to its large size. *Hystrix indica* (Indian crested porcupine) and long eared *Hemiechinus collaris* (Desert hedgehog) have same suffix ‘say’. Indian crested porcupine is known as ‘kanday wali say’ due to long spines while long eared desert hedgehog is known as ‘chotay kanday ali say’ because of small spines.

Based on habitat some mammals were named as ‘fasli choha’ (*M. meltada*) lives in cultivated fields, whereas ‘jungli choha (*T. indica*) is found in forests only. Likewise, *Lepus nigricollis dayanus* (Desert hare) lives in forest and is named jungli khargush or saya, while *Oryctolagus cuniculus* (Domestic rabbit) lives in houses and is known as khargush or saya. Five species of mammals were noted to have more than one local names viz. desert hare ‘jungli saya and jungli khargush’, Indian wild boar (*Sus scrofa*) ‘baarla and soor’ and domestic rabbit ‘khargush and saya’. Saya and baarla are common names in the forest land and rural areas, whereas khurgush and soor are used in urban areas. Chotay kanday ali say and Kandyari Choha are common names of *Hemiechinus collaris* Pangolin and Sipple are also common names of *Manis crassicaudata* in all areas (Table 2).

**Table 2:** Cultural uses of mammals and birds in the study area

S. no	Scientific, local & common name	MCU	FM	RFM	Med. use	Cultural uses									
						Mag	Entt	Do	To	Cc	Pt	Or	Fo	Na	Ha
	Mammals														
1.	*Bos taurus* L. Cow, Gay	5	36	0.33	✓	X	X	✓	X	✓	X	✓	✓	X	X
2.	*Bubalus bubalis* L. Buffalo, Mujh	5	40	0.37	✓	X	X	✓	X	✓	X	✓	✓	X	X
3.	*Camelus dromedaries* L. Camel, Ount	5	24	0.22	✓	✓	X	✓	X	✓	X	X	✓	X	X
4.	*Canis aureus* L. Asiatic jackal, Gidar	3	17	0.16	✓	X	X	X	X	X	X	✓	X	X	✓
5.	*Canis lupus familiaris* L. Dog, Kuta	5	19	0.17	✓	X	✓	X	X	X	✓	X	X	✓	✓
6.	*Capra aegagrus hircus* L. Goat, Bakri	5	32	0.29	✓	X	X	✓	X	✓	X	✓	✓	X	X
7.	*Equus africanus* von Heuglin Donkey, Gadha	5	22	0.2	✓	X	X	✓	X	✓	X	✓	X	X	X
8.	*Equus caballus* L. Horse, Kurrah	5	28	0.26	✓	X	✓	✓	X	✓	X	✓	X	X	X
9.	*Felis chaus* Schreber Jungle cat, Jungli billi	3	15	0.14	✓	X	X	X	X	X	X	✓	X	X	✓
10.	*Felis domesticus* L. Cat, Billi	3	46	0.42	✓	X	X	X	X	X	✓	X	X	✓	X
11.	*Funnambulus pennanti* Wroughton Northern palm squirrel, Gulahri	1	7	0.06	✓	X	X	X	X	X	X	X	X	X	X
12.	*Hemiechinus collaris* Gray Long eared desert hedgehog Chotay kanday ali say, Kandyari Choha	2	11	0.1	✓	X	X	X	✓	X	X	X	X	X	X
13.	*Herpestes javanicus* E. Geoffroy Small Indian mongoose, Neola	3	12	0.11	✓	X	✓	X	X	X	X	✓	X	X	X
14.	*Homo sapiens* L. Human, Insan	1	9	0.08	✓	X	-	X	X	X	X	X	X	X	X
15.	*Hystrix indica* Kerr Indian crested porcupine, Kanday wali say	4	52	0.48	✓	✓	X	X	✓	X	X	✓	X	X	X
16.	*Lepus nigricollis dayanus* F. Cuvier Desert hare, Jungli saya, Jungli khargush	4	54	0.5	✓	X	✓	X	X	X	X	✓	✓	X	X
17.	*Manis crassicaudata* E. Geoffroy Indian Pangolin, Pangolin, Sipple	1	8	0.07	✓	X	X	X	X	X	X	X	X	X	X
18.	*Millardia meltada* Gray Soft-furred field rat, Fasli Choha	1	6	0.06	✓	X	X	X	X	X	X	X	X	X	X
19.	*Mus musculus* L. House mouse, Chota Choha	1	4	0.04	✓	X	X	X	X	X	X	X	X	X	X
20.	*Nesokia indica* Gray Short tailed mole rat, Chhoti push wala choha	1	3	0.03	✓	X	X	X	X	X	X	X	X	X	X
21.	*Oryctolagus cuniculus*l. Domestic rabbit, Khargush, Saya	4	14	0.13	✓	X	X	✓	X	X	X	✓	✓	X	X
22.	*Ovis aries* L. Sheep, Bairh	5	23	0.21	✓	X	X	✓	X	✓	X	✓	✓	X	X
23.	*Pteropus giganteus* Brunnich Indian flying fox bat, Chamgadar	1	10	0.09	✓	X	X	X	X	X	X	X	X	X	X
24.	*Rattus rattus* L. House rat, Wada Choha	1	5	0.05	✓	X	X	X	X	X	X	X	X	X	X
25.	*Suncus etruscus* Savi Mediterranean pygmy shrew, Choti chachondar	1	2	0.02	✓	X	X	X	X	X	X	X	X	X	X
26.	*Suncus murinus* L. House shrew, Waddi chachondar	1	2	0.02	✓	X	X	X	X	X	X	X	X	X	X
27.	*Sus scrofa* L. Indian wild boar, Baarla, Soor	4	20	0.18	✓	X	✓	X	X	X	X	X	X	✓	✓
28.	*Tatera indica* Hardwicke Indian gerbil, Jungli Choha	1	3	0.03	✓	X	X	X	X	X	X	X	X	X	X
29.	*Ursus thibetanus G. Cuvier* Bear, Richh	7	26	0.24	✓	✓	✓	X	✓	✓	X	✓	X	X	✓
30.	*Vulpes bengalensis* Shaw Indian/Bengal fox, Lomri	3	13	0.12	✓	X	X	X	X	X	X	✓	X	X	✓
	Birds														
31.	*Acridotheres ginginianus* Latham Bank Myna, Shark	3	21	0.193	✓	X	X	X	X	X	X	✓	X	X	X
32.	*Acridotheres tristis* L. Common Myna, Lali	2	3	0.028	X	X	X	X	X	X	X	✓	X	X	X
33.	*Acrocephalus dumetorum* Blyth Blyth’s Reed Warbler, Dabh peeddi	2	6	0.055	X	X	X	X	X	X	X	✓	X	X	X
34.	*Acrocephalus melanopogon* Temminck Moustached Sedge Warbler, Chhoti Peeddi	2	4	0.037	X	X	X	X	X	X	X	✓	X	X	X
35.	*Alauda arvensis* L Eurasian Lark, Chandol	4	35	0.321	X	X	✓	X	X	X	X	✓	✓	X	X
36.	*Alauda gulgula* Franklin Small Skylark, Chhota chandol	4	35	0.321	X	X	✓	X	X	X	X	✓	✓	X	X
37.	*Alcedo atthis* L. Common Kingfisher, Chhota machhera	2	12	0.11	X	X	X	X	X	X	X	✓	X	X	X
38.	*Amandava amandava* L. Red Munia, Lal moonia	2	11	0.101	X	X	X	X	X	X	X	✓	X	X	X
39.	*Amaurornis phoenicurus* Pennant, White-breasted Waterhen, Chitthikki jal kukri	4	25	0.229	X	X	✓	X	X	X	X	✓	✓	X	X
40.	*Anas clypeata* L. Shoveler, Balchi	2	9	0.083	X	X	X	X	X	X	X	✓	X	X	X
41.	*Anas crecca* L. Common Teal, Til	2	3	0.028	X	X	X	X	X	X	X	✓	X	X	X
42.	*Anas penelope* L. Eurasian Wigeon. Wijan	4	5	0.046	X	X	✓	X	X	X	X	✓	✓	X	X
43.	*Anas platyrhynchos domesticus* L. Domestic Duck, Batakh	5	55	0.505	✓	X	X	✓	X	✓	X	✓	✓	X	X
44.	*Anas platyrhynchos* L. Mallard, Nilsir	3	11	0.101	✓	X	X	X	X	X	X	✓	X	X	X
45.	*Anas querquedula* L. Garganey, Nili til	3	22	0.202	X	X	X	X	X	X	X	✓	V	X	X
46.	*Anas strepera* L. Gadwall, Gaidwal	2	4	0.037	X	X	X	X	X	X	X	✓	X	X	X
47.	*Anhinga melanogaster* Pennant Snake Bird, Bhujanga	2	6	0.055	X	X	X	X	X	X	X	✓	X	X	X
48.	*Anser indicus* Latham Bar-headed Goose, Sawa magh	4	22	0.202	X	X	✓	X	X	X	X	✓	✓	X	X
49.	*Anthus campestris* L. Tawny Pipit, Baggi charchari	2	4	0.037	X	X	X	X	X	X	X	✓	X	X	X
50.	*Anthus novaeseelandiae* Gmelin Richard Pipit, Charchari	2	2	0.018	X	X	X	X	X	X	X	✓	X	X	X
51.	*Anthus trivialis* L. Tree Pipit, Rukh charchari	2	6	0.055	X	X	X	X	X	X	X	✓	X	X	X
52.	*Anus auta* L. Pintail Duck, Sinkhpur	4	38	0.349	X	X	✓	X	X	X	X	✓	✓	X	X
53.	*Apus affinis* Gray Little Swift, Chhoti ateran	2	11	0.101	X	X	X	X	X	X	X	✓	X	X	X
54.	*Aquila rapax* Temminck Tawny Eagle, Chhota baaz	3	9	0.083	✓	X	X	X	X	X	X	✓	X	X	X
55.	*Ara macao* L. Macaw, Macaw	3	24	0.22	✓	X	X	✓	X	X	X	✓	X	X	X
56.	*Ardea cinerea* L. Grey Heron, Nari	2	15	0.138	X	X	X	X	X	X	X	✓	X	X	X
57.	*Ardea cinerea* L. Purple Heron, Kirmachi nari	2	17	0.156	X	X	X	X	X	X	X	✓	X	X	X
58.	*Ardeola grayii* Sykes Indian Pond Heron, Chhappari bagla	2	2	0.018	X	X	X	X	X	X	X	✓	X	X	X
59.	*Athene brama* Temminck Spotted Little Owlet, Ullo	5	31	0.284	✓	✓	X	X	X	X	X	✓	X	✓	X
60.	*Aythya ferina* L. Common Pochard, Pochad	4	35	0.321	X	X	✓	X	X	X	X	✓	✓	X	X
61.	*Aythya fuligula* L. Tufted Duck, Bodal murgabi	4	34	0.312	X	X	✓	X	X	X	X	✓	✓	X	X
62.	*Bubulcus ibis* L. Cattle Egret, Badami bagla	3	6	0.055	✓	X	X	X	X	X	X	✓	X	X	X
63.	*Buteo buteo* L. Common Buzzard, Tisa	2	2	0.018	X	X	X	X	X	X	X	✓	X	X	X
64.	*Buteo rufinus* Cretzschmar Long-legged Buzzard, Chuhamar tisa	2	3	0.028	X	X	X	X	X	X	X	✓	X	X	X
65.	*Calandrella brachydactyla*Leisler, Greater Short-toed Lark, Chandol	4	35	0.321	X	X	✓	X	X	X	X	✓	✓	X	X
66.	*Calidris alpine* L. Tateri	2	5	0.046	X	X	X	X	X	X	X	✓	X	X	X
67.	*Calidris minuta* Leisler Little Stint, Panlawa	2	5	0.046	X	X	X	X	X	X	X	✓	X	X	X
68.	*Calidris temminckii* Leisler Temminck’s Stint	2	5	0.046	X	X	X	X	X	X	X	✓	X	X	X
69.	*Caprimulgus europaeus* L. European Nightjar, Chapaki	2	27	0.248	X	X	X	X	X	X	X	✓	X	X	X
70.	*Carpodacus erythrinus* Pallas Common Rosefinch, Lal tooti	4	15	0.138	X	X	✓	X	X	X	X	✓	✓	X	X
71.	*Centropus sinensis* Stephens Common Crow Pheasant, Jal Kukar	3	18	0.165	✓	X	X	X	X	X	X	✓	X	X	X
72.	*Cercomela fusca* Blyth Common Rock chat, Lal galri	2	3	0.028	X	X	X	X	X	X	X	✓	X	X	X
73.	*Ceryle rudis* L. Small Pied kingfisher, Kilkila	2	9	0.083	X	X	X	X	X	X	X	✓	X	X	X
74.	*Charadrius alexandrinus* L. Snowy Plover, Kalarwala marwa	3	17	0.156	✓	X	X	X	X	X	X	✓	X	X	X
75.	*Chlidonias hybridus* Pallas Whiskered Tern, Taheri	2	4	0.037	X	X	X	X	X	X	X	✓	X	X	X
76.	*Chrysomma altirostre* JerdonSind Babbler, Serhari	2	2	0.018	X	X	X	X	X	X	X	✓	X	X	X
77.	*Cisticola juncidis* Rafinesque Fan-tailed Warbler, Phanka Peeddi	2	4	0.037	X	X	X	X	X	X	X	✓	X	X	X
78.	*Clamator jacobinus* Boddaert Pied Crested Cuckoo, Koail	2	14	0.128	X	X	X	X	X	X	X	✓		X	X
79.	*Columba livia* Gmelin Blue Rock Pigeon, Jangli kabotar	6	60	0.55	✓	X	✓	X	X	✓	X	✓	✓	X	X
80.	*Coracias benghalensis* L. Indian Roller, Nil kanth	2	3	0.028	X	X	X	X	X	X	X	✓	X	X	X
81.	*Coracias garrulus* L. Kashmir Roller, Nil Kanth	2	8	0.073	X	X	X	X	X	X	X	✓	X	X	X
82.	*Corvus splendens* Vieillot House Crow, Kaan	4	28	0.257	✓	X	X	X	X	X	X	✓	X	✓	X
83.	*Coturnix coturnix* L. Common Quail, Batera	6	58	0.532	✓	X	✓	X	X	✓	X	✓	✓	X	X
84.	*Cursorius coromandelicus* Gmelin, Indian Courser, Nukri	2	4	0.037	X	X	X	X	X	X	X	✓	X	X	X
85.	*Dendrocitta vagabunda* Latham Indian Tree Pie, Chhota kaan, Lagoja	2	5	0.046	X	X	X	X	X	X	X	✓	X	X	X
86.	*Dicrurus macrocercus* Vieillot Black Drongo, Japal kalchit, Chepu	2	5	0.046	X	X	X	X	X	X	X	✓	X	X	X
87.	*Egretta alba* L. Large Egret, Wadda bagla	3	10	0.092	✓	X	X	X	X	X	X	✓	X	X	X
88.	*Egretta garzetta* L. Little Egret, Bauna bagla	3	8	0.073	✓	X	X	X	X	X	X	✓	X	X	X
89.	*Egretta intermedia* Wagler Intermediate Egret, Gabhla bagla	3	12	0.11	✓	X	X	X	X	X	X	✓	X	X	X
90.	*Elanus caeruleus* Desfontaines Black Winged Kite, Chiti ail	2	10	0.092	X	X	X	X	X	X	X	✓	X	X	X
91.	*Emberiza bruniceps* Brandt Red-headed Bunting, Lal sir booli	2	25	0.229	X	X	X	X	X	X	X	✓	X	X	X
92.	*Emberiza schoeniclus* L. Reed Bunting, Booli	2	14	0.128	X	X	X	X	X	X	X	✓	X	X	X
93.	*Eremopterix grisea* Scopoli Ashy Crowned Finch lark, Saleti sir chandol	4	35	0.321	X	X	✓	X	X	X	X	✓	✓	X	X
94.	*Eudynamys scolopacea* L. Koel, Koal	4	24	0.22	X	X	✓	X	X	X	X	✓	✓	X	X
95.	*Falco tinnunculus* L. Eurasian Kestrel, Lal shikra	2	11	0.101	X	X	X	X	X	X	X	✓	X	X	X
96.	*Falco chicquera* Daudin, Red Necked Falcon, Lal-gardan baaz	2	10	0.092	X	X	X	X	X	X	X	✓	X	X	X
97.	*Ficedula parva* Bechstein Red-breasted Flycatcher, Lal gala tik tiki	2	4	0.037	X	X	X	X	X	X	X	✓	X	X	X
98.	*Francolinus francolinus* L. Black partridge, Kala tittar	6	56	0.514	✓	✓	✓	X	X	X	X	✓	✓	X	X
99.	*Francolinus pondicerianus* Gmelin, Indian Grey Partridge, Bhura tittar	5	23	0.211	X	✓	✓	X	X	X	X	✓	✓	X	X
100.	*Fulica atra* L. Eurasian Coot, Koot	2	11	0.101	X	X	X	X	X	X	X	✓	X	X	X
101.	*Gallicrex cinerea* Gmelin Watercock, Jal murgha	2	13	0.119	X	X	X	X	X	X	X	✓	X	X	X
102.	*Gallinula chloropus* L. Common Moorhen, Jal kukri	4	34	0.312	X	X	✓	X	X	X	X	✓	✓	X	X
103.	*Gallus gallus* L. Domestic Chicken, Murghi	5	62	0.569	✓	X	X	✓	X	✓	X	✓	✓	X	X
104.	*Gelochelidon nilotica* Gmelin Gull-billed Tern, Bularh taheri	2	5	0.046	X	X	X	X	X	X	X	✓	X	X	X
105.	*Grus grus* L. Common Crane, Waddi kunj	2	9	0.083	X	X	X	X	X	X	X	✓	X	X	X
106.	*Halcyon smyrnensis* L. White-throated Kingfisher, Wadda machhera	2	14	0.128	X	X	X	X	X	X	X	✓	X	X	X
107.	*Hieraaetus fasciatus* Sibley & Monroe Bonnelli’s Eagle, Baaz	3	14	0.128	✓	X	X	X	X	X	X	✓	X	X	X
1108.	*Himantopus himantopus* L. Black-winged Stilt, Lam latta	2	15	0.138	X	X	X	X	X	X	X	✓	X	X	X
109.	*Hippolais caligata* Lichtenstein Booted Warbler, Chita gala Peeddi	2	7	0.064	X	X	X	X	X	X	X	✓	X	X	X
110.	*Hirundo rustica* L. Barn or Common Swallow, Ababil	2	5	0.046	X	X	X	X	X	X	X	✓	X	X	X
111.	*Hirundo smithii* Leach Wire-tailed Swallow, Tar punjha	2	4	0.037	X	X	X	X	X	X	X	✓	X	X	X
112.	*Hoplopterus indicus* Boddaert Red-wattled Lapwing, Tatihri	2	13	0.119	X	X	X	X	X	X	X	✓	X	X	X
113.	*Ixobrychus sinensis* Gmelin Yellow Bittern, Bora bagla	2	4	0.037	X	X	X	X	X	X	X	✓	X	X	X
114.	*Larus fuscus* L Lesser Black-headed Gull, Chhota damra	2	4	0.037	X	X	X	X	X	X	X	✓	X	X	X
115.	*Lonchura malabarica* L. Indian Silverbill	4	17	0.156	X	X	✓	X	X	X	X	✓	✓	X	X
116.	*Lymnocryptes minimus* Brünnich, Jack Snipe, Rangla chaha	2	6	0.055	X	X	X	X	X	X	X	✓	X	X	X
117.	*Meleagris gallopavo* L. Turkey, Turkey	5	30	0.275	✓	X	X	✓	X	✓	X	✓	✓	X	X
118.	*Merops orientalis* Latham Little Green Bee-eater, Chhota path ranga	2	7	0.064	X	X	X	X	X	X	X	✓	X	X	X
119.	*Merops supercilliosus* L. Blue-cheeked Bee-eater, Chhota path ranga	3	5	0.046	✓	X	X	X	X	X	X	✓	X	X	X
120.	*Milvus migrans migrans* Boddaert, Indian Kite, Cheil, Ail	2	8	0.073	X	X	X	X	X	X	X	✓	X	X	X
121.	*Motacilla alba alboides* Hodgson, Hodgeson’s Pied Wagtail, Wadda mamola	2	3	0.028	X	X	X	X	X	X	X	✓	X	X	X
122.	*Motacilla alba dukhunensis* Sykes, Siberian Pied Wagtail, Wadda mamola	2	3	0.028	X	X	X	X	X	X	X	✓	X	X	X
123.	*Motacilla cinerea* Tunstall Grey Wagtail, Slati mamola	2	4	0.037	X	X	X	X	X	X	X	✓	X	X	X
124.	*Motacilla citreola calcarata* PallasYellow-headed Black-backed Wagtail, Pila kala Mamola	2	6	0.055	X	X	X	X	X	X	X	✓	X	X	X
125.	*Motacilla citreola citreola* Pavlova Yellow-Headed Black-Collared Wagtail, Pila Mamola	2	4	0.037	X	X	X	X	X	X	X	✓	X	X	X
126.	*Motacilla citreola werae* Pavlova Yellow-headed Grey-backed Wagtail Pila si mamaloa	2	4	0.037	X	X	X	X	X	X	X	✓	X	X	X
127.	*Motacilla maderaspatensis* Gmelin, Large Pied Wagtail, Wada mamola	2	3	0.028	X	X	X	X	X	X	X	✓	X	X	X
128.	*Mycteria leucocephala* Pennant Painted Stork, Chitra lamdhing	2	11	0.101	X	X	X	X	X	X	X	✓	X	X	X
129.	*Nectarinia asiatica* Latham Purple Sunbird, Kala pidda, Shakar khora	2	7	0.064	X	X	X	X	X	X	X	✓	X	X	X
130.	*Nycticorax nycticorax* L. Night Heron, Chor bagla	2	7	0.064	X	X	X	X	X	X	X	✓	X	X	X
131.	*Oenanthe isabellina* Temminck, Isabelline Wheatear, Kali akha wheater	3	15	0.138	✓	X	X	X	X	X	X	✓	X	X	X
132.	*Oenanthe picata* Blyth Eastern Wheatear, Kali cheeti wheatear	3	20	0.183	✓	X	X	X	X	X	X	✓	X	X	X
133.	*Oriolus oriolus* L. Golden Oriole, Pilak	2	13	0.119	X	X	X	X	X	X	X	✓	X	X	X
134.	*Orthotomus sutorius* Pennant Tailor Bird, Derzi	2	3	0.028	X	X	X	X	X	X	X	✓	X	X	X
135.	*Parus major* L. Great Tit, Wadda tit	2	8	0.073	X	X	X	X	X	X	X	✓	X	X	X
136.	*Passer domesticus* L. House Sparrow, Chiri	6	64	0.587	✓	X	✓	X	X	✓	X	✓	✓	X	X
137.	*Passer hispaniolensis* Temminck, Willow Sparrow, Chini chiri	2	15	0.138	X	X	X	X	X	X	X	✓	X	X	X
138.	*Pavo cristatus* L. Peacock, Moor	3	25	0.229	✓	X	X	✓	X	X	X	✓	X	X	X
139.	*Pericrocotus ethologus* Bangs & Phillips Long-tailed Minivet, Lam punjhi saheli	2	7	0.064	X	X	X	X	X	X	X	✓	X	X	X
140.	*Pernis ptilorhynchus* Temminck, Crested Honey Buzzard, Makhi tissa	2	4	0.037	X	X	X	X	X	X	X	✓	X	X	X
141.	*Phalacrocorax niger* Vieillot Little Cormorant, Jal kaan	2	3	0.028	X	X	X	X	X	X	X	✓	X	X	X
142.	*Phoenicurus ochruros* Gmelin Black Redstart, Kala thirthara	2	4	0.037	X	X	X	X	X	X	X	✓	X	X	X
143.	*Phylloscopus subviridis* Brooks Brooks’s Leaf Warbler, Hari peeli Peeddi	2	4	0.037	X	X	X	X	X	X	X	✓	X	X	X
144.	*Ploceus philippinus* L. Baya Weaver, Bijra	2	3	0.028	X	X	X	X	X	X	X	✓	X	X	X
145.	*Porzana parva* Scopoli Little Crake, Jal bater	2	15	0.138	X	X	X	X	X	X	X	✓	X	X	X
146.	*Prinia burnesii* Blyth Long-tailed Grass Warbler, Bori Peeddi	2	3	0.028	X	X	X	X	X	X	X	✓	X	X	X
147.	*Prinia gracilis* Lichtenstein Streaked Long-tailed Warbler, Lumbi push Peeddi	2	5	0.046	X	X	X	X	X	X	X	✓	X	X	X
148.	*Prinia inornata* Sykes Tawny Prinia, Chhoti bori Peeddi	4	27	0.248	X	X	✓	X	X	X	X	✓	✓	X	X
149.	*Prinia socialis* Sykes Ashy long-tailed Warbler, Uchi push Peeddi	2	3	0.028	X	X	X	X	X	X	X	✓	X	X	X
150.	*Psittacula eupatria* L. Large Indian Parakeet, Wada tota	4	50	0.459	X	X	X	X	X	✓	✓	✓	X	X	X
151.	*Psittacula krameri* Scopoli Rose-ringed Parakeet, Gani wala Tota	5	50	0.459	X	X	X	X	X	X	✓	✓	X	X	X
152.	*Pycnonotus cafer* L. Red-vented Bulbul, Pahari bulbul	2	2	0.018	X	X	X	X	X	X	X	✓	X	X	X
153.	*Pycnonotus leucogenys* Gray White-cheeked Bulbul, Bulbul	2	2	0.018	X	X	X	X	X	X	X	✓	X	X	X
154.	*Rallus aquaticus* L. Water Rail	2	13	0.119	X	X	X	X	X	X	X	✓	X	X	X
155.	*Recurvirostra avosetta* L. Pied Avocet, Chaha	2	3	0.028	X	X	X	X	X	X	X	✓	X	X	X
156.	*Remiz pendulinus* L. Penduline Tit, Tit	2	6	0.055	X	X	X	X	X	X	X	✓	X	X	X
157.	*Rhipidura aureola* Lesson White-browned Fantail Flycatcher Phanka tik tiki	2	3	0.028	X	X	X	X	X	X	X	✓	X	X	X
158.	*Riparia paludicola* Vieillot Indian Sindh Martin, Martin	2	35	0.321	X	X	X	X	X	X	X	✓	X	X	X
159.	*Riparia riparia* L. Collard Sand Martin, Martin ababil	2	4	0.037	X	X	X	X	X	X	X	✓	X	X	X
160.	*Rynchops albicollis* Swainson Indian Skimmer, Pancheera	2	4	0.037	X	X	X	X	X	X	X	✓	X	X	X
161.	*Saxicola leucura* Blyth White-tailed Bushchat, Galri	2	5	0.046	X	X	X	X	X	X	X	✓	X	X	X
162.	*Saxicoloides fulicata* L. Indian Robin, Kalla Peedda	2	3	0.028	X	X	X	X	X	X	X	✓	X	X	X
163.	*Sterna acuticauda* Gray Black-bellied Tern, Kali chonge taheri	2	5	0.046	X	X	X	X	X	X	X	✓	X	X	X
164.	*Sterna albifrons* Pallas Little Tern, Choti taheri	2	6	0.055	X	X	X	X	X	X	X	✓	X	X	X
165.	*Sterna aurantia* Gray Indian River Tern, Dariai taheri	2	4	0.037	X	X	X	X	X	X	X	✓	X	X	X
166.	*Streptopelia decaocto* Frivaldszky Indian Ring Dove, Kogi, Ghogi	6	45	0.413	✓	X	✓	X	X	X	X	✓	✓	✓	X
167.	*Streptopelia orientalis* Latham Oriental turtle Dove, Totru	6	44	0.404	✓	X	✓	X	X	X	X	✓	✓	✓	X
168.	*Streptopelia senegalensis* L. Little Brown Dove, Chhoti tutru, Chhoti kogi	6	36	0.33	✓	X	✓	X	X	X	X	✓	✓	✓	X
169.	*Streptopelia tranquebarica* Hermann, Red Turtle Dove, Lal totru	6	47	0.431	✓	X	✓	X	X	X	X	✓	✓	✓	X
170.	*Sturnus roseus* L. Rosy Starling, Gulabi tilyar, Gulabi maina	2	4	0.037	X	X	X	X	X	X	X	✓	X	X	X
171.	*Sturnus vulgaris* L. Common Starling, Tilyar, Maina	2	5	0.046	X	X	X	X	X	X	X	✓	X	X	X
172.	*Sylvia curruca* L. Lesser Whitethroat, Chitt kanthi peeddi	2	4	0.037	X	X	X	X	X	X	X	✓	X	X	X
173.	*Tachybaptus ruficollis* Pallas Little Grebe, Dubkian	2	5	0.046	X	X	X	X	X	X	X	✓	X	X	X
174.	*Tadorna ferruginea* Pallas Common Shelduck, Surkhab	2	3	0.028	X	X	X	X	X	X	X	✓	X	X	X
175.	*Tadorna tadorna* L. Ruddy Shelduck, Surmai	2	6	0.055	X	X	X	X	X	X	X	✓	X	X	X
176.	*Tephrodornis pondicerian* Gmelin, Common Wood Shrike, Latora	2	6	0.055	X	X	X	X	X	X	X	✓	X	X	X
177.	*Tringa glareola* L. Wood Sandpiper	2	5	0.046	X	X	X	X	X	X	X	✓	X	X	X
178.	*Tringa nebularia* Gunnerus Greenshank, Hara chaha	2	4	0.037	X	X	X	X	X	X	X	✓	X	X	X
179.	*Tringa ochropus* L. Green Sandpiper	2	6	0.055	X	X	X	X	X	X	X	✓	X	X	X
180.	*Tringa stagnatilis* Bechstein Marsh Sandpiper	2	5	0.046	X	X	X	X	X	X	X	✓	X	X	X
181.	*Turdoides caudatus* Dumont Common Babbler, Serhari	2	4	0.037	X	X	X	X	X	X	X	✓	X	X	X
182.	*Turdoides earlei* Blyth Striated Babbler, Dharidar serhari	2	7	0.064	X	X	X	X	X	X	X	✓	X	X	X
183.	*Turdoides striatus* Dumont Jungle Babbler, Jangli serhari	2	9	0.083	X	X	X	X	X	X	X	✓	X	X	X
184.	*Upupa epops* L. Common Hoopoe, Hud-hud	2	4	0.037	X	X	X	X	X	X	X	✓	X	X	X
185.	*Vanellus vanellus* L. Great Plover, Waddi karvank	2	4	0.037	X	X	X	X	X	X	X	✓	X	X	X

*MCU* (Medicinal and Cultural Uses), *FM* (Frequency of Mention), *RFM* (Relative Frequency of Mention), *Med* (Medicinal), *Mag* (Magic), *Entt* (Entertainment), *Do* (Domestic), *To* (Tool), *Cc* (Commercial), *Pt* (Pet), *Or* (Ornamental), *Fo* (Food), *Na* (Narrative), *Ha* (Harmful)

The local name of 96.2% bird species are mentioned (Table 2). However, local name of 3.8% species including *Rallus aquaticus*, *Calidris temminckii*, *Tringa stagnatilis, Tringa ochropus, Tringa glareola* and *Lonchura malabarica* could not be searched. Around 8 bird species were noted to have more than one local name. These include: *Milvus migrans migrans,* (Cheil and Ail), *Streptopelia decaocto* (Kogi and Ghogi), *Streptopelia orientalis* (Tutru and Chhoti kogi), *Nectarinia asiatica* (Kala pidda and Shaker khora), *Dicrurus macrocercus,* (Japal kalchit and Chepu), *Sturnus vulgaris* (Tilyar and Maina), *Sturnus roseus* (Gulabi tilyar and Gulabi maina), and *Dendrocitta vagabunda,* (Chhota kaan and Lagoja). About 5.2% species have synonyms; because of their resemblance with other bird species such as *Merops orientalis,* and *Merops supercilliosus* have synonym chhota path ranga; the synonym of *Oenanthe isabellina, Oenanthe picata* is wheatear; *Coracias garrulus* and *Coracias benghalensis* have synonym nil kanth; while *Chrysomma altirostre,* and *Turdoides caudatus* called as serhari.

Interestingly, the vernacular names of 26 bird species were associated with their voice. These species were: *Phalacrocorax niger* (jal kaan), *Anas Penelope* (wijan), *Milvus migrans* (ail), *Elanus caeruleus* (chiti ail), *Francolinus francolinus* (kala tittar), *Coturnix coturnix* (batera), *Grus grus* (waddi kunj), *Recurvirostra avosetta* (chaha), *Hoplopterus indicus* (tatihri), *Calidris alpine* (tateri), *Tringa nebularia* (hara chaha), *Gelochelidon nilotica* (bularh taheri), *Chlidonias hybridus* (taheri), *Streptopelia orientalis* (Totru), *Psittacula eupatria* (wada tota), *Psittacula krameri,*(ganiwala tota), *Clamator jacobinus* (koail), *Eudynamys scolopacea* (koal), *Ceryle rudis* (kilkila), *Upupa epops* (hud-hud), *Coracias benghalensis* (nil kanth), *Hirundo rustica* (ababil), *Anthus campestris* (baggi charchari), *Corvus splendens* (kaan), *Carpodacus erythrinus* (lal tooti) and *Athene brama* (ullo).

The local name and English name of 10.3% species were same. Such as Teal for (*Anas crecca*), Gadwall (*Anas strepera*), Wigeon (*Anas Penelope*), Pochard (*Aythya ferina*), Coot (*Fulica atra*), Koel (*Eudynamys scolopacea*), Martin (*Riparia paludicola*), Tit (*Remiz pendulinus* & *Parus major*), Bulbul (*Pycnonotus* spp.), Macaw (*Ara macao*), Wheatear (*Oenanthe* spp.) and Turkey (*Meleagris gallopavo*). This may be due the fact that, English is the official language of Pakistan and British Government had ruled over this region more than 9 decades.

### Body part(s) used

**Fig. 2:**
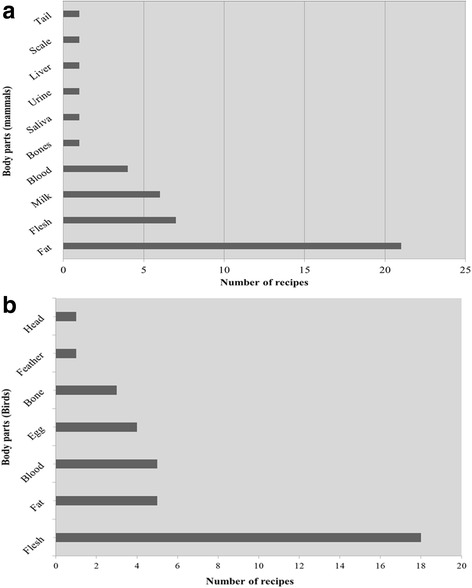
Body parts of mammals (**a**) and birds (**b**) used in various recipes

The body parts of mammals and birds species used in different recipes are presented in Fig. 2a and b. In mammals, fat was the most utilized body part (21 recipes), followed by flesh (7), milk (6) and blood (4), while remaining parts were used in one recipe only. Among birds, flash was the most commonly used body part with maximum application of 18 recipes, followed by fat and blood (each in 5 recipes), egg (4 recipes) and bones (3 recipes).

The inhabitants of the study area use fat and flesh to treat skin infections, rheumatic pains, burning sensation, body swelling and as sex stimulant. The presence of omega-3 fatty acid in fat that reduces inflammation may involve treating human ailments [37]. As this compound is also useful in neurological disorder, atherosclerosis, thrombotic and aging affects [38–40]. Likewise, milk of *Bubalus bubalis* (Buffalo), *Bos taurus* (Cow), *Capra aegagrus hircus* (Goat), *Camelus dromedaries* (Camel), *Equus africanus* (Donkey) and *Ovis aries* (Sheep) is used to treat muscular pain, weakness, fever, and as sexual tonic. This may be due to the presence of high contents of proteins, lipids, vitamins and minerals in milk, which strengthens the body, reduce joint pain and increase sexual potency [41–45].

Blood of different species such as donkey, domestic rabbit, desert hare, camel, spotted little owlet, cattle egret, large egret, little egret and intermediate egret was effective in abdominal dropsy, arthritis, burning sensation, sexual weakness and dysentery. Flesh of different mammals and birds was used to cure asthma, epilepsy, joint pain, sexual debility and skin infections. Human’s urine was reported against herpes and to treat ear pain in the study area. It has been documented that the urine of cow, sheep, camel, hyrax, goat, rhinoceros and ass effective in the treatment of disinfection, skin diseases, syphilis, tuberculosis, asthma, mouth infection, foot diseases, chronic ailment, acne, back pain, fever, anemia, nervous problem, memory loss, as antifungal, throat, rashes, burn, ear and eyes infections [2, 20, 46–56]. In addition, urine of camel inhibits cell proliferation, enhance apoptosis, maintain cyclin-dependent kinase inhibitor p21[48], and has high resistance against heat as well as fungal diseases [54].

### Ethnomedicinal uses of mammals and birds

**Fig. 3:**
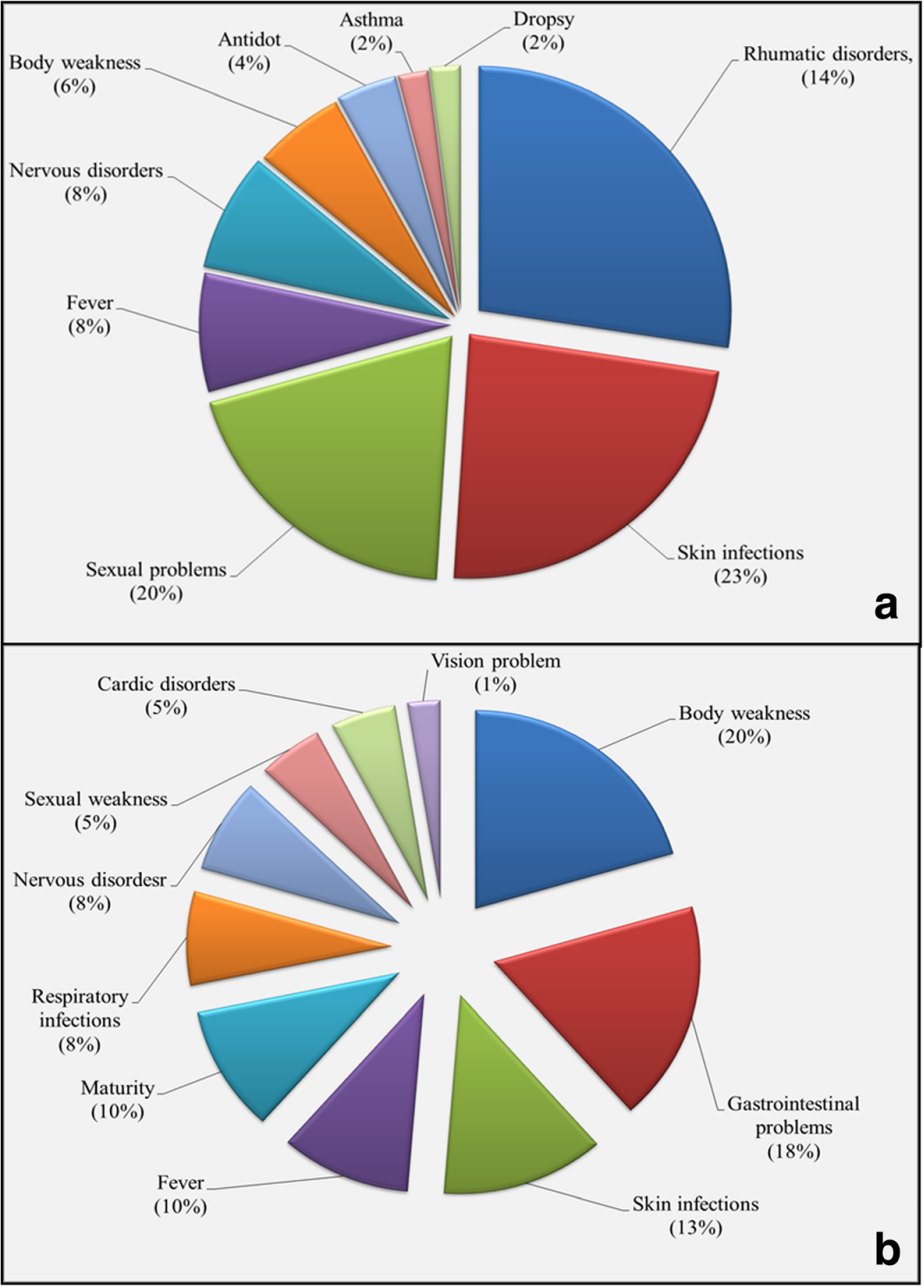
Percentage of diseases curd by using mammals (**a**) and birds (**b**) species

Present investigation is the first report on ethnomedicinal uses and cultural values of mammals and bird species in Pakistan. The inhabitants of the study area use different animals to treat health disorders and possess significant traditional knowledge particularly on medicinal and cultural uses of mammals and birds species. In total, 30 mammalian and 28 bird species are used to treat various diseases in the study area (Table 3). The Fig. 3a and b demonstrates percentage of animal based (mammals and birds) recipes used to treat various diseases by the inhabitants of the study area. Rheumatic disorders, skin infections, sexual weakness and gastrointestinal disorders were among the topmost ailments treated, followed be body pain, burning sensation and paralysis. In mammals 23% recipes were used to treat skin infections, followed by sexual problems and rheumatic disorders (20 and 14%, respectively), whereas for birds highest percentage recipes were used to treat body weakness, gastrointestinal disorders and skin infections (20, 18 and 13%, respectively). Nutritional deficiency, lack of hygienic environment and social evils may attribute in high prevalence of these diseases in the study area.

**Table 3:** Medicinal uses of mammals and birds and their comparison with previous reports

S. no	Scientific, common and local name	Part used	Application	Diseases cured	Previous reports	Reference	SI	IMA	FL	RPL	ROP
	Mammals										
1.	*Bos taurus* L. Cow, Gay	Fat, milk, Flesh	Topical and oral	Feet wounds, body pain, fever, poison effect	Fever, bone fever, memory loss, paralysis, asthma, stomach ache, gastritis, diarrhea, eye infection, tuberculosis, pesticide	[46, 47, 59, 62, 65, 77, 78]	0.08	36	100	1	100
2.	*Bubalus bubalis* L. Buffalo Mujh	Fat, Milk, flesh	Topical and oral	Feet wound, body pain, fever, poison effect	Pain, wound, jaundice, ascites, rheumatic pain, weakness, osteoporosis, thrombosis	[18, 55–57, 59, 61, 77, 79]	0.18	16	40	1	40
3.	*Camelus dromedaries* L. Camel Ount	Milk, blood	Topical and oral	Muscular pain, weakness, arthritis	Acidity, hepatitis B and C	[60, 64]	0	10	42	0.89	37
4.	*Canis aureus* L. Asiatic jackal Gidar	Flesh, bones	Topical	Skin diseases	Asthma, sciatica, arthritis, body pain, gout, skin diseases, paralysis	[46, 47, 57, 70, 78]	0.14	11	65	0.63	41
5.	*Canis lupus familiaris* L. Dog Kuta	Fat, flesh	Topical	Sexual power	Weakness, poison, fever	[77]	0	8	42	0.7	30
6.	*Capra aegagrus hircus* L. Goat Bakri	Milk	Oral	Increase sexual efficiency	Fever, eye tonic, tonsillitis, asthma, tuberculosis, menstrual disorder, toothache, anemia, dysentery, bronchitis, jaundice, diarrhea, blindness	[55, 58–62, 64]	0	13	41	1	41
7.	*Equus africanus* von Heuglin & Fitzinger Donkey Gadha	Milk, blood	Topical	Abdominal dropsy, arthritis	Arthritis, madness, abdominal dropsy, tuberculosis	[55, 58, 70]	0.5	9	41	0.81	33
8.	*Equus caballus* L. Horse Kurrah	Fat	Topical	Skin infection	Rabies, skin diseases, burn, allergy, arthritis, body pain, neuralgia, osteoporosis	[50, 55, 56]	0.13	11	39	1	39
9.	*Felis chaus* Schreber Jungle cat Jungli billi	Fat	Topical	Joint Pain	Leucoderma	[55]	0	10	67	0.56	37
10.	*Felis domesticus* L. Cat Billi	Fat	Topical	Rheumatic pain, skin infections	Fever, arthritis	[57, 58]	0	46	100	1	100
11.	*Funnambulus pennanti* Wroughton Northern palm squirrel Gulahri	Flesh	Topical and oral	Epilepsy	Epilepsy	[59]	1	4	57	0.26	15
12.	*Hemiechinus collaris* Gray Long eared desert hedgehog Chotay kanday ali say, Kandyari Choha	Fat	Topical	Rheumatic pain, body ache			0	7	64	0.41	26
13.	*Herpestes javanicus* E. Geoffroy Saint-Hilarie Small Indian mongoose Neola	Fat	Topical	Sexual power			0	8	67	0.44	30
14.	*Homo sapiens* L. Human Insan	Saliva, urine	Topical	Herpes, ear pain	Eye infections, wound, hiccup	[49–51, 58, 70, 77, 80]	0	6	67	0.33	22
15.	*Hystrix indica* Kerr Indian crested porcupine Kanday wali say	Fat	Topical	Skin infection, Rheumatic pain			0	26	50	1	50
16.	*Lepus nigricollis dayanus* F. Cuvier Desert hare Jungli saya, Jungli khargush	Flesh, liver, blood	Topical and oral	Asthma, burning sensation, paralysis	Tonic, chicken pox, wheezing, stomach and joint pain, high blood pressure, asthma	[46, 47, 55, 56, 59, 70, 77, 78]	0.13	27	50	1	50
17.	*Manis crassicaudata* E. Geoffroy Indian Pangolin Pangolin, Sipple	Scale, flesh	Topical	Feet swelling, Sexual power	Feet swelling, piles, blood pressure, head ach, asthma, anti-haemorrhoidal, warts, ear pain, angina	[55, 56, 59, 65, 66, 70]	0.1	3	38	0.3	11
18.	*Millardia meltada* Gray Soft-furred field rat Fasli Choha	Fat	Topical	Joint pain			0	3	50	0.22	11
19.	*Mus musculus* L. House mouse Chota Choha	Fat	Topical	Enhancement of semen	Arthritis, analgesic	[60, 80]	0	2	50	0.15	7
20.	*Nesokia indica* Gray Short tailed mole rat Chhoti push wala choha	Fat	Topical	Joint pain			0	2	67	0.11	7
21.	*Oryctolagus cuniculus*L. Domestic rabbit Khargush, Saya	Tail, blood	Topical	Burning sensation, weakness	Bronchial diseases, stomachache	[63, 64]	0	14	100	0.52	52
22.	*Ovis aries* L. Sheep Bairh	Fat, milk, flesh	Topical and oral	Skin burn and crack, weakness, joint pain	Edema, fractures, joint pain, sterility, flu, skin burn and crack, muscular pain, swellings, weakness,	[20, 45, 47, 52, 56, 80–82]	0.2	23	100	0.85	85
23.	*Pteropus giganteus* Brunnich Indian flying fox bat Chamgadar	Fat	Topical	Body and backbone pain, sexual power	Asthma, bronchitis	[55, 56, 61, 77]	0	5	50	0.37	19
24.	*Rattus rattus* L. House rat Wada Choha	Fat	Topical	Joint pain	Convulsions, semen enhancement, wounds healing,	[56, 57, 61, 69]	0	3	60	0.19	11
25.	*Suncus etruscus* Savi Mediterranean pygmy shrew Choti chachondar	Fat	Topical	Scrotal swelling			0	1	50	0.07	4
26.	*Suncus murinus* L. House shrew Waddi chachondar	Fat	Topical	Scrotal swelling	Snake bite, scrotal swelling	[56, 61]	0.5	1	50	0.07	4
27.	*Sus scrofa* L. Indian wild boar Baarla, Soor	Fat	Topical	Paralysis, burn	Inflammatory, joint pain, fracture, paralysis, burn, snake bite, fever, piles, cough, cold, anti-haemorrhoidal, warts, earache, angina	[46, 47, 50, 55, 56, 59, 60, 62, 63, 65, 70, 77, 80]	0.17	10	50	0.74	37
28.	*Tatera indica* Hardwicke Indian gerbil Jungli Choha	Fat	Topical	Lumbago			0	1	33	0.11	4
29.	*Ursus thibetanus* G. Cuvier Bear Richh	Fat	Topical	Sexual power			0	7	27	0.96	26
30.	*Vulpes bengalensis* Shaw Indian/Bengal fox Lomri	Fat	Topical	Epilepsy			0	9	69	0.48	33
	Birds										
31.	*Acridotheres tristis* L. Common Myna, Lali	Flesh	Oral	Whooping cough, weakness			0	15	71.43	0.66	47
32.	*Anas platyrhynchos domesticus* L. Domestic Duck, Batakh	Egg	Oral	Weak eye-side, weakness, low blood pressure		[20, 55, 56, 69]	0	55	100.	1.00	100
33.	*Anas platyrhynchos* L. Mallard, Nilsir	Flesh, egg	Oral	Paralysis, weakness	Erectile dysfunction, scarlet fever, body strength, weakness		0.2	10	90.91	0.34	31
34.	*Aquila rapax* Temminck Tawny Eagle, Chhota baaz	Fat	Topical	Breast swelling	Chest pain	[45]	0	4	44.44	0.28	13
35.	*Ara macao* L. Macaw, Macaw	Fat	Topical	Pneumonia			0	9	37.50	0.75	28
36.	*Athene brama* Temminck Spotted Little Owlet, Ullo	Blood	Topical	Sexual weakness	Rickets, cough	[55]	0	18	58.06	0.97	56
37.	*Bubulcus ibis* L. Cattle Egret, Badami bagla	Blood, Flesh	Topical and oral	Dysentery			0	1	16.67	0.19	3
38.	*Centropus sinensis* Stephens Common Crow Pheasant, Jal Kukar	Flesh	Oral	Body-ache, weakness			0	5	27.78	0.56	16
39.	*Charadrius alexandrinus* L. Snowy Plover, Kalarwala marwa	Egg	Oral	Typhoid		[47, 55–57, 60, 63, 64, 69]	0	6	35.29	0.53	19
40.	*Columba livia* Gmelin Blue Rock Pigeon, Jangli kabotar	Flesh, Feather	Oral	Paralysis	Menorrhagia, Bronchitis, puberty in young girls, paralysis, epilepsy, anemia, infertility	[55, 83]	0.17	53	88.33	1.00	88
41.	*Corvus splendens* Vieillot House Crow, Kaan	Bone	Topical	For ear infection	Lethargy, aphrodisiac, anemia, body aches, stomach disorder		0	4	14.29	0.88	13
42.	*Coturnix coturnix* L. Common Quail, Batera	Head of the bird, flesh	Oral	Enhance memory, improve sexual power	Skin diseases, anemia, body weakness, enhance memory power	[47, 55, 56]	0.25	30	51.72	1.00	52
43.	*Egretta alba* L. Large Egret, Wadda bagla	Blood, Flesh	Topical and oral	Dysentery			0	5	50.00	0.31	16
44.	*Egretta garzetta* L. Little Egret, Bauna bagla	Blood, Flesh	Topical and oral	Dysentery	Asthma, body strength, breathing trouble, immune enhancer	[55, 56]	0	2	25.00	0.25	6
45.	*Egretta intermedia* Wagler Intermediate Egret, Gabhla bagla	Blood, Flesh	Topical and oral	Dysentery			0	7	58.33	0.38	22
46.	*Francolinus francolinus* L. Black partridge, Kala tittar	Flesh and Bone soup	Oral	Bronchitis, weakness	Bronchitis	[64]	0.5	24	42.86	1.00	43
47.	*Gallus gallus* L. Domestic Chicken, Murghi	Egg, flesh	Oral	Fever, weakness, low blood pressure	Sprains, strains, nourishing food, eye-each, bronchitis, diabetes, burst furuncles, asthma, Indigestion, sinusitis, shortness of breath, bronchitis, nervous problems, rheumatism, stuffy nose, weak bones, flu, weakness, sore throat, furuncle, burns, night blindness, eye infection, evil eye	[20, 47, 49, 50, 58, 61–63, 78, 82, 84]	0	62	100	1.00	100
48.	*Hieraaetus fasciatus* Sibley & Monroe Bonnelli’s Eagle, Baaz	Fat	Topical	Breast swelling	Breast swelling	[58]	1	3	21.43	0.44	9
49.	*Meleagris gallopavo* L. Turkey, Turkey	Flesh	Oral	Asthma			0	10	33.33	0.94	31
50.	*Oenanthe isabellina* Temminck, Isabelline Wheatear, Kali akha wheater	Fat	Topical	Gastric problems in infants			0	8	53.33	0.47	25
51.	*Oenanthe picata* Blyth Eastern Wheatear, Kali cheeti wheatear	Fat	Topical	Gastric problems in infants			0	2	10	0.63	6
52.	*Passer domesticus* L. House Sparrow, Chiri	Flesh	Oral	Weakness, fever	Increase sexual desire, aphrodisiac, allergy, paralysis, impotency, gas trouble, constipation, Chickenpox,	[47, 56, 62, 64, 84]	0	64	100	1.00	100
53.	*Pavo cristatus* L. Peacock, Moor	Bone	Topical	Wound, pus	Blurred vision, anemia, Abscess, eye diseases, body strength, ear infection, hiccup, asthma	[55, 56, 62, 65]	0	7	28.00	0.78	22
54.	*Streptopelia decaocto* Frivaldszky Indian Ring Dove, Kogi, Ghogi	Flesh	Oral	Maturity in girls	Early maturity in girls	[64]	1	13	28.89	1.00	29
55.	*Streptopelia orientalis* Latham Oriental turtle Dove, Totru	Flesh	Oral	Maturity in girls			0	13	29.55	1.00	30
56.	*Streptopelia senegalensis* L. Little Brown Dove, Chhoti tutru, Chhoti kogi	Flesh	Oral	Maturity in girls			0	13	36.11	1.00	36
57.	*Streptopelia tranquebarica* Hermann, Red Turtle Dove, Lal totru	Flesh	Oral	Maturity in girls			0	13	27.66	1.00	28
58.	*Upupa epops* L. Common Hoopoe, Hud-hud	Flesh	Oral	Kidney problems	Gall bladder stone	[84]	0	1	25.00	0.13	3

*SI* (Similarity Index), *IMA* (Informants of Major Ailment), *FL* (Fidelity Level), *RPL* (Relative Popularity Level), *ROP* (Rank order priority)

Local people use body fat of *Felis domesticus* (Cat) to treat skin infections and rheumatic pain. These findings were in agreement to Benarjee, Srikanth [57] and andHaileselasie [58]. Milk of *C. aegagrus hircus* (Goat) is used as sexual tonic. However, different parts of same species have been reported to cure fever, eye tonic, tonsillitis, asthma, tuberculosis, irregular menstrual cycle, toothache, anemia, dysentery, bronchitis, jaundice, diarrhea, anemia and blindness [55, 56, 58–62]. According to local inhabitants, milk of *C. dromedaries* (Camel) is highly effective in the treatment of sexual weakness and muscular pain, whereas tail and blood of *O. cuniculus* (Domestic rabbit) are useful against burning sensation and weakness. Same species have been reported to treat acidity, bronchial disease, stomach disorder, hepatitis B and C [60, 63, 64]. Scales and flesh of *M. crassicaudata* (Indian pangolin) were used in the treatment of feet swelling and as sexual tonic, respectively. Same species is used to treat piles, blood pressure, headache, asthma [55, 56, 59, 61, 65, 66].

The ethnomedicinal uses of *C. dromedaries* (Camel), *C. aegagrus hircus* (Goat), *Canis lupus familiaris* (Dog), *Felis chaus* (Jungli cat), *F. domesticus* (Cat), *H. collaris* (Long eared desert hedgehog), *Herpestes javanicus* (Small Indian mongoose), *Homo sapiens* (Human), *H. indica* (Indian crested porcupine), *M. meltada* (Soft-furred field rat), *M. musculus* (House mouse), *Nesokia indica* (Short tailed mole rat), *O. cuniculus* (Domestic rabbit), *R. rattus* (House rat), *S. estruscus* (Mediterranean pygmy shrew), *Tatera indica* (Indian gerbil) and *Ursus thibetanus* (Bear) were reported for the first time (Table 3). In addition, these species exhibited zero similarity index with previous literature. Inhabitants of the study area use these species to treat sexual power, rheumatic pain, herpes, lumbago, burning sensation, enhancement of semen, ear pain, skin infections, muscular pain, weakness, and arthritis. Some species i.e. *Funnambulus pennanti* (Northern palm squirrel), *E. africanus* (Donkey), *S. murinus* (House shrew), and *O. aries* (Sheep) exhibited maximum similarity index with previous studies (1, 0.5, 0.5 and 0.2, respectively).Due to illegal hunting and extensive use in traditional medicines Indian pangolin is at verge of extinction and has been included in “Red Listed” species by International Union for Conservation of Nature (IUCN) [67, 68].

Only, 28 species of birds out of 155 were used in traditional medicines by the inhabitants of the study area (Table 3). The ethnomedicinal uses of *Acridotheres tristis*, *Anas platyrhynchos domesticus*, *Aquila rapax*, *Ara macao*, *Athene brama*, *Bubulcus ibis*, *Charadrius alexandrinus*, *Corvus splendens*, *Centropus sinensis*, *Egretta alba*, *Egretta garzetta*, *Egretta intermedia*, *Gallus gallus*, *Meleagris gallopavo*, *Oenanthe isabellina*, *Oenanthe picata*, *Passer domesticus, Pavo cristatus*, *Streptopelia orientalis*, *Streptopelia senegalensis, Streptopelia tranquebarica* and *Upupa epops* have not been reported before and exhibited 0 similarity Index. These species were reported against respiratory disorders (asthma, pneumonia, and cough), cardiovascular disorders, skin infections (swelling, wounds, pus, and ear infection), sexual weakness, typhoid, body-ache, fever, gastric problems, maturity in girls and kidney problems.

*Anas platyrhynchos* was used for the treatment of paralysis, weakness. Same species was reported to treat erectile dysfunction, scarlet fever, body strength and weakness, showed 0.2 similarity index [19, 20, 55, 56, 69]. *Columba livia*, was used to treat paralysis and have 0.17 similarity index with previous reports [46, 47, 57, 60, 63, 64, 69]. Local inhabitants use *Coturnix coturnix* to enhance memory, improve sexual power. Same species has been reported against skin diseases, anemia, body weakness, enhance memory power and its similarity index is 0.25 [47, 55, 56]. *Hieraaetus fasciatus* and *Streptopelia decaocto* were used for the treatment of the breast swelling and early maturity in young girls respectively and have highest similarity index 1.

### Cultural uses

**Fig. 4:**
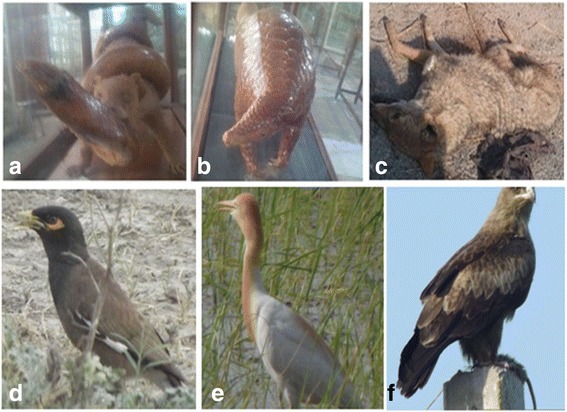
Some important mammals and bird species of the study area. **a** Stuffed mongoose with stuff snake. **b** Indian pangolin (**c**) Indian Jackal (**d**), common Myna (**e**) Egret (**f**) Tawny eagle

The cultural uses of mammals and bird species are given in Table 2. Spines of *H. indica* (Indian crested porcupine) were used in magic or superstitions; however presence of spines creates disgusting among the people that may leads to clash. Likewise, hairs and bones of *U. thibetanus* (Bear) and *C. dromedaries* (Camel) were used to treat black magic (Kala Jadoo). Six mammals’ species were used for enjoyment of the people such as dog fight, mongoose contest with snake, bear and horse dance, hunting of desert hare and Indian wild boar. Dogs are commonly used for hunting of desert hare and Indian wild boar. Horses with decorated craft (Baggi) are used in wedding ceremony. *B. bubalis* (Buffalo), *B. tarus* (Cow), *C. aegagrus hircus* (Goat), *C. dromedaries* (Camel), *E.* *africanus* (Donkey), *Equus caballus* (Horse), *Oryctolagus cuniculus* (Domestic rabbit) and *O. aries* (sheep) are reared for milk and milk products (curd, butter, ghee), meet, leather and wool. Skin of large and medium size mammals species were used to make leather products. Hairs of *Canis aureus*, *C. aegagrus hircus, E. caballus, F. chaus, H. indica, Herpestes javanicus*, *O. aries*, *O. cuniculus* and *Vulpes bengalensis* were used in stuffed toys (Fig 4). These findings were in agreement to del Valle, Naranjo [16].

Spines of *H. indica* and *H. collaris* were used as needles while bones of *U. thibetanus* were used as a defensive tool. Bear (*Ursus thibetanus*) are not present in the wild areas of Areas surrounding the river Chenab are not natural habitat of *U. thibetanus*, however body parts of this species are imported from Azad Jammu and Kashmir and Northern areas of Pakistan. Sun dried dung of *B. bubalis* and *B. gaurus* is used for heating purpose and to cook food. Likewise, local inhabitants used to train dogs for hunting desert hare and Indian wild boar. Ten percent of the reported species were linked with traditional narrated stories or superstitions such as; people of the area thought that if cat (*F. domesticus*) crossed ahead of any person during journey, then it would be inauspicious. Spiritually and socially it is believed, that Allah (God) may not accept prayer of a person that speaks the name of Soor (*S. scrofa*). Similarly, presence of the dog (*C. lupus familiaris*) in the house may stop the blessing of Allah (God). According todel Valle, Naranjo [16]79% mammals species in Playon de la Gloria, 50% in Reforma Agraria, 47% in Naha and 42% in Metzabok-Mexico were supposed to be harmful.

In the study sites, people eat specific birds, as they obey the rules of Islam. Among birds, 17.4% species (herbivore, granivore, frugivore and omnivore which do not eat dead animals) were edible and used as food Table 2, while scavengers, carnivores, insectivore and piscivore are prohibited to eat in Islam. Local hunters mimic the voices of doves, partridges and quails. They use golara (birds in cage) to attracts other species of birds. Punjabi net trap and mist net are also used to capture the live birds. Previous results showed that wild birds used as a source of food in many areas of the world i.e. India [60, 70]; Pakistan [64]; Philippines [71]; Brazil [72, 73].

Six birds were linked with narrative stories, such as the voice of crow is thought to be an indication of guest. Similarly, the presence of owl is supposed to be infamy in home; arrival or presence of doves (Indian ring dove, red turtle dove, little brown dove and Oriental turtle dove) in house linked with the influx of prosperity. Many magicians used owlet blood and carcasses for magic. These findings were almost same as reported [74] in Punjab, Pakistan.

About 96.8% of reported bird species are wild, while 3.2% are domesticated. People of the study area like to keep Parakeets (Large Indian Parakeet and Rose-ringed Parakeet) as a pet bird. Eight species of the birds were used commercially. Such as common quail farming is growing day by day. Fried meet of common quail, house sparrow and blue rock pigeon is very delicious. Parakeet’s species are used commercially for the lottery. Domestic chicken, duck and turkey are kept in home and at farms for the meat purposes. About 15.5% species were used for hunting or entrainment and all reported birds were used for the ornamental purposes; because they are stuffed by local people and their feathers are used in making mud toys.

### Relative frequency of mention (RFM)

The animal species, which are reported by the maximum number of informants are frequently used to treat various diseases, exhibited high relative frequency of mention (RFM) ranged from 0.02 to 0.587 (Table2). Among mammals *Lepus nigricollis dayanus* (Desert hare) had maximum RFM (0.50), followed by *Hystrix indica* (Indian crested porcupine) and *Felis domesticus* (Cat) (0.48 and 0.42, respectively). Whereas lowest RFM value (0.02) was calculated in *Suncus estruscus* (Mediterranean pygmy shrew) and *Suncus murinus* (House shrew). Among birds: *Passer domesticus* (House Sparrow) depicted highest RFM value (0.587), while *Gallus gallus* (Domestic chicken) and *Columba livia* (Blue Rock Pigeon) were ranked second and third with RFM value of 0.569 and 0.550, respectively.

### Fidelity level (FL)

**Fig. 5:**
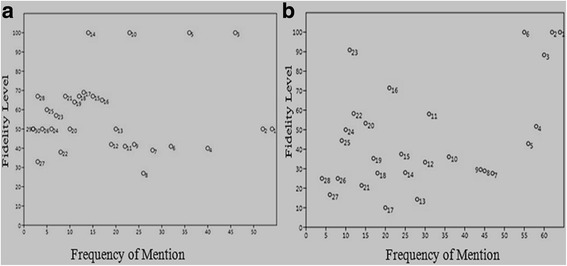
The relationship between informant numbers and the number of that mammalian (**a**) and avian (**b**) species application; circled numbers showed the mammal and birds names as given in Table 2

Fidelity level (FL) is used to identify species that are most preferred by the inhabitants for the treatment of certain ailments. Animal species with topmost medicinal uses in a particular area have maximum fidelity level [75, 76]. In the present investigation fidelity level of mammals and birds species varied from 10 to 100% (Table 2). *B. taurus* Smith (Cow), *F. domesticus* (Desert hare’)*, Oryctolagus cuniculus* (Domestic rabbit) and *Ovis aries* (Sheep) were the mammals species, which depicted 100% FL, while *Ursus thibetanus* (Bear) showed lowest FL percentage (27%) as mentioned in (Fig 5). Fat, milk and flesh of these species were used to treat skin infections, fever, rheumatic pain, and to reduce poisonous effects. Among birds; *Anas platyrhynchos domesticus* (Domestic duck)*, Gallus gallus* (Domestic chicken) and *Passer domesticus* (House sparrow) exhibited 100% FL. Beside this, six species of birds depicted more than 70% FL, which include: *Anas platyrhynchos* (90.91%)*, Columba livia* (88.33%) and *Acridotheres ginginianus* (71.43%) S2B Fig*.* The FL of mammals and bird species were calculated for the first time. Therefore, these species could be used for in depth chemical profiling and to investigate pharmaceutical properties, which may confirm their medicinal worth.

### Relative popularity level (RPL)

**Fig. 6:**
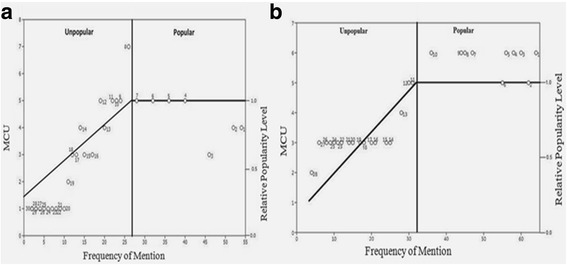
The relationship between informant numbers and the percentage of informants who argued similar use for that mammals (**a**) and Birds (**b**); circled numbers show mammalian and bird names as they present in Table 2

The Relative popularity level (RPL) of mammals and bird species are given in Table 3. Approximately, 7 species of mammals that depicted highest importance were included for further discussion. For the mammals species cited by 2 to 26 informants (Fig. 6a), the frequency of use per mammal increases linearly with increase in the frequency of mention (y-1.5 + 0.130×; correlation coefficient *r* = 0.661). Conversely, the half number of uses for those species mentioned by 27 informants or more does not increase with the increased FM. All mammals species mentioned by less than 27 informants (23 mammals species) were therefore classified as unpopular, whereas those cited by 27 informants or more (7 mammals species) are classified as popular. The *B. bubalis* (buffalo), *B. taurus* (cow)*, C. aegagrus hircus* (goat), *E. caballus* (horse), *F. domesticus* (cat), *H. indica* (Indian crested porcupine) and *L. nigricollis dayanus* (desert hare) were the most popular mammals with 1.0 RPL value.

In birds, 10 species received more attention by informants, therefore included for further discussion (Fig 6b). The bird species cited by 4 to 64 informants, number of uses per bird increases with the increase in the number of informants (*r* = 0.71). The popular bird species with 1.000 RPL value were; *P. domesticus, G. gallus, C. livia, C. coturnix, F. francolinus, A. platyrhynchos domesticus, S. tranquebarica, S. decaocto, S. orientalis* and *S. senegalensis.* These findings were comparable with Friedman, Yaniv [35] and Ali-Shtayeh, Yaniv [36]. Furthermore, high popularity of mammalian and bird species might be attributed to wider geographic distribution, informant’s awareness and cultural knowledge.

### Rank order priority (ROP)

The healing potential of each mammal and bird species was documented using its FL values, while ROP is used to give appropriate rank to species with different FL values. The RPL of each species derived from Fig 6a and b ; was used as correction factor to adjust the FL values. The measured level of rank order priority (ROP) of each mammal and bird species is mentioned in Table 3. The ROP value of only four mammal species out of 30 and 4 bird species out of 28 was above 50. The *B. taurus* (Cow) and *F. domesticus* (Cat) were highly utilized with maximum ROP = 100, followed by *O. aries* (Sheep) and *O. cuniculus* (Domestic rabbit) have ROP (85 and 52, respectively). Among, birds ROP value of *P. domesticus* and *G. gallus* was 100 and that of *C. livia* was 88. Decrease in ROP value may be due to decreasing popularity of medicinal and cultural uses of animals among indigenous peoples. Additionally, the informants of the rural areas have more information and interaction with cultural and medicinal uses of mammals and birds compared to urban areas. These findings were analogous to previous results for medicinal species of Negev district [35] and Palestinian area [36].

## Conclusion

Inhabitants of the study area showed strong association with surrounding fauna and possess significant traditional knowledge particularly on mammals and birds species. In the present study, the ethnomedicinal and cultural uses of; 30% mammals and 46% birds’ species were reported for the first time. Moreover, 33% mammals and 79% birds’ species depicted zero similarity Index. These findings could be helpful for conservation and sustainable use of animal biodiversity in the region. Further investigation to screen pharmacological active substances and in vitro*/*in vivo valuation of biological activities in mammals and birds’ species with maximum FL and FM could be significant in animal based drug discoveries.

## References

[1] Kaplan H, Hill K, Lancaster J, Hurtado AM (2000). A theory of human life history evolution: diet, intelligence, and longevity. Evol Anthr.

[2] Alves RR, Rosa IL, Neto NAL, Voeks R (2012). Animals for the gods: magical and religious faunal use and trade in Brazil. Hum Ecol.

[3] Marques JGW (1994). A fauna medicinal dos índios Kuna de San Blas (Panamá) ea hipótese da universalidade zooterápica.

[4] Bagde NS, Hampa J (2013). An ethnozoological studies and medicinal values of vertebrate origin in the adjoining areas of Pench National Park of Chhindwara District of Madhya Pradesh, India. Ind Int J Life Sci.

[5] Alves RR, Rosa IL (2005). Why study the use of animal products in traditional medicines?. J Ethnobiol Ethnomed.

[6] Santos-Fita D, Costa-Neto E, Cano-Contreras E, Costa Neto E, Santos Fitas D, Vargas CM. El quehacer de la etnozoología. Manual de Etnozoología. 2009:23–44.

[7] Londoño-Betancourth JC (2009). Valoración cultural del uso e importancia de la fauna silvestre en cautividad en tres barrios de Pereira (Risaralda)*.* Boletín Científico. Centro de Museos. Museo Hist Nat.

[8] Kang S, Phipps MJ, Asia TE (2003). A question of attitude: South Korea's traditional medicine practitioners and wildlife conservation.

[9] Marques J (1997). Fauna medicinal: Recurso do ambiente ou ameaça à biodiversidade. Mutum.

[10] Berlin B. Ethnobiological Classification: Principles of Categorization of Plants and Animals in Traditional Societies. New Jersey: Princeton University Press; 2014.

[11] Baumeister RF. The cultural animal: Human nature, meaning, and social life. New York: Oxford University Press: 2005.

[12] Rosegrant MWH, azell PB (2000). Transforming the rural Asian economy: The unfinished revolution.

[13] Milton K (2003). The critical role played by animal source foods in human (Homo) evolution. J Nutr.

[14] Kamanga P, Vedeld P, Sjaastad E (2009). Forest incomes and rural livelihoods in Chiradzulu District, Malawi. Ecol.l Econ.

[15] Turbay S, Ulloa A (2002). Aproximaciones a los estudios antropológicos sobre la relación entre el ser humano y los animales*.* Rostros culturales de la fauna: las relaciones entre los humanos y los animales en el contexto colombiano.

[16] del Valle YG, Naranjo EJ, Caballero J, Martorell C, Ruan-Soto F, Enríquez PL (2015). Cultural significance of wild mammals in mayan and mestizo communities of the Lacandon Rainforest, Chiapas, Mexico. J Ethnobiol Ethnomed.

[17] Ruan-Soto F, Caballero J, Martorell C, Cifuentes J, González-Esquinca AR, Garibay-Orijel R (2013). Evaluation of the degree of mycophilia-mycophobia among highland and lowland inhabitants from Chiapas. Mexico J Ethnobiol Ethnomed.

[18] Alves RR, Rosa IL (2007). Zootherapy goes to town: The use of animal-based remedies in urban areas of NE and N Brazil. J Ethnopharmacol.

[19] Alves RRN (2012). Relationships between fauna and people and the role of ethnozoology in animal conservation. Ethnobiol Conserv..

[20] Alves RRN, Neta ROS, Trovão D, Barbosa J, Barros AT, Dias TLP (2012). Traditional uses of medicinal animals in the semi-arid region of northeastern Brazil. J Ethnobiol Ethnomed.

[21] Mesquita GP, Barreto LN. Evaluation of mammals hunting in indigenous and rural localities in Eastern Brazilian Amazon. Ethnobiol Conserv. 2015;4:2. doi:10.15451/ec2015-1-4.2-1-14.

[22] Roberts TJ (1997). The Mammals of Pakistan.

[23] Mirza ZB, Wasiq H (2007). A field guide to birds of Pakistan.

[24] Siddiqi TA, Tahir-Kheli S (2004). Water and Security in South Asia.

[25] Sheikh MS. Punjab G Go, editor. District Pre-Investment Study 2012, vol. 2012. p. 1–376.

[26] Sheikh MS. Punjab GGO, editor. District Pre-Investment Study 2012, vol. 2012. p. 1–28.

[27] Sheikh MS, Punjab SGO (2012). District Pre-Investment Study 2012.

[28] Umair M, Ilyas U, Altaf M (2013). Diversity and Ecology of Parthenium weeds ar head khanki.

[29] Roberts TJ (2005). Field guide to the large and medium-sized mammals of Pakistan.

[30] Roberts TJ (2005). Field guide to the small mammals of Pakistan.

[31] Roberts TJ, The Birds of Pakistan. Vol I Place Oxford; University Press: 1991.

[32] Roberts TJ, The Birds of Pakistan. Vol II Place Oxford; University Press: 1992.

[33] Tardío JPardo-de-Santayana M. (2008). Cultural importance indices: a comparative analysis based on the useful wild plants of Southern Cantabria (Northern Spain) 1. Econ Bot.

[34] Alexiades MN, Sheldon JW (1996). Selected guidelines for ethnobotanical research: a field manual.

[35] Friedman J, Yaniv Z, Dafni A, Palewitch D (1986). A preliminary classification of the healing potential of medicinal plants, based on a rational analysis of an ethnopharmacological field survey among Bedouins in the Negev Desert Israel. J Ethnopharmacol.

[36] Ali-Shtayeh MS, Yaniv Z, Mahajna J (2000). Ethnobotanical survey in the Palestinian area: a classification of the healing potential of medicinal plants. J Ethnopharmacol.

[37] Wilson L. Fats and oils for optimum health. The Center for Development. 2015;

[38] Breteler MM (2000). Vascular risk factors for Alzheimer’s disease:: An epidemiologic perspective. Neurobiol Aging.

[39] Kalmijn S (2000). Fatty acid intake and the risk of dementia and cognitive decline: a review of clinical and epidemiological studies. J Nutr Health Aging.

[40] Haag M (2003). Essential fatty acids and the brain. Can J Psychiatr.

[41] Hemme T, Otte J, Echeverri Perico R, Paarlberg R, Walker I, Pino H, Horton D, Polanía Vorenberg J, Toro Calderón J, López Balmaceda C (2010). Status and prospects for smallholder milk production.

[42] Alabdulkarim B (2012). Effect of camel milk on blood glucose, cholesterol, triglyceride and liver enzymes activities in female Albino rats. World Appl Sci J.

[43] Sabahelkhier M, Faten M, Omer F (2012). Comparative Determination of Biochemical Constituents between Animals (Goat, Sheep, Cow and Camel) Milk with Human Milk. Res J Recent Sci.

[44] Contarini G, Povolo M (2013). Phospholipids in milk fat: composition, biological and technological significance, and analytical strategies. Int J Mol Sci.

[45] Vats R, Thomas S (2015). A study on use of animals as traditional medicine by Sukuma Tribe of Busega District in North-western Tanzania. J Ethnobiol Ethnomed.

[46] Lohani U (2011). Eroding ethnozoological knowledge among Magars in Central Nepal. Indian JTrad Knowl.

[47] Lohani U (2011). Traditional uses of animals among jirels of Central Nepal. Ethno Med.

[48] Al-Yousef N, Gaafar A, Al-Otaibi B, Al-Jammaz I, Al-Hussein K, Aboussekhra A (2012). Camel urine components display anti-cancer properties in vitro. J Ethnopharmacol.

[49] Barros FB, Varela SA, Pereira HM, Vicente L (2012). Medicinal use of fauna by a traditional community in the Brazilian Amazonia. J Ethnobiol Ethnomed.

[50] Kim H, Song MJ (2013). Ethnozoological study of medicinal animals on Jeju Island. Korea J Ethnopharmacol.

[51] Kim H, Song MJ (2014). Analysis of ethnomedicinal practices for treating skin diseases in communities on Jeju Island (Korea). Indian J Trad Knowl..

[52] Melo R, Silva O, Souto A, Alves RRN, Schiel N (2014). The role of mammals in local communities living in conservation areas in the Northeast of Brazil: an ethnozoological approach. Trop Conserv Sci.

[53] Mohanty I, Senapati MR, Jena D, Palai S (2014). Diversified uses of cow urine. Intern J Pharm Pharmaceut Sci.

[54] Al-Awadi A, Al-Judaibi A (2015). Effects of Heating and Storage on the Antifungal Activity of Camel Urine. Clin Microbiol.

[55] Vijayakumar S, Prabhu S, Yabesh JM, Prakashraj R (2015). A quantitative ethnozoological study of traditionally used animals in Pachamalai hills of Tamil Nadu. India J Ethnopharmacology.

[56] Vijayakumar S, Yabesh JM, Prabhu S, Ayyanar M, Damodaran R (2015). Ethnozoological study of animals used by traditional healers in Silent Valley of Kerala. India J Ethnopharmacol.

[57] Benarjee G, Srikanth K, Ramu G, Ramulua K (2010). Ethnozoological study in a tropical wildlife sanctuary of Eturunagaram in the Warangal district, Andhra Pradesh. Ind J Trad Knowled.

[58] Haileselasie TH (2012). Traditional zootherapeutic studies in Degu’a Tembien, Northern Ethiopia. Cur Res J Biol Sci.

[59] Dixit A, Kadavul K, Rajalakshmi S, Shekhawat M (2010). Ethno-medico-biological studies of South India. Indian J Trad Knowl..

[60] Jaroli D, Mahawar MM, Vyas N (2010). An ethnozoological study in the adjoining areas of Mount Abu wildlife sanctuary. India J Ethnobiol Ethnomed.

[61] Chellappandian M, Pandikumar P, Mutheeswaran S, Paulraj MG, Prabakaran S, Duraipandiyan V, Ignacimuthu S, Al-Dhabi N (2014). Documentation and quantitative analysis of local ethnozoological knowledge among traditional healers of Theni district, Tamil Nadu. India J Ethnopharmacolo.

[62] Bagde N, Jain S (2015). Study of traditional man-animal relationship in Chhindwara District Of Madhya Pradesh. India J Glob Bioscie.

[63] Alonso-Castro AJ, Carranza-Álvarez C, Maldonado-Miranda JJ, del Rosario J-SM, Quezada-Rivera DA, Lorenzo-Márquez H, Figueroa-Zúñiga LA, Fernández-Galicia C, Ríos-Reyes NA, de León-Rubio MÁ (2011). Zootherapeutic practices in Aquismón, San Luis Potosí México. J Ethnopharmacol.

[64] Arshad M, Ahmad M, Ahmed E, Saboor A, Abbas A, Sadiq S (2014). An ethnobiological study in Kala Chitta hills of Pothwar region, Pakistan: multinomial logit specification. J Ethnobiol Ethnomed.

[65] Mishra N, Rout S, Panda T (2011). Ethno-zoological studies and medicinal values of Similipal Biosphere Reserve, Orissa India. African J Pharm and Pharmacol.

[66] Kulkarni BD (2011). Folk therapies of Katkaries from maharashtra. Indian J Trad Knowl.

[67] Mohapatra RK, Panda S, Acharjyo L, Nair M, Challender DW (2015). A note on the illegal trade and use of pangolin body parts in India. Traffic Bull.

[68] Zhou Z-M, Zhou Y, Newman C, Macdonald DW (2014). Scaling up pangolin protection in China. Frontiers Ecol Environ.

[69] Mootoosamy A, Mahomoodally MF (2014). A quantitative ethnozoological assessment of traditionally used animal-based therapies in the tropical island of Mauritius. J Ethnopharmacol.

[70] Chinlampianga M, Singh RK, Shukla AC (2013). Ethnozoological diversity of Northeast India: Empirical learning with traditional knowledge holders of Mizoram and Arunachal Pradesh. Indian J Tradit Knowl.

[71] Van der Ploeg JVan Weerd M. Agta bird names: an ethno-ornithological survey in the Northern Sierra Madre Natural Park Philippines. Forktail. 2010:127–31.

[72] Alves RRN, Leite RCL, Souto WMS, Bezerra DM, Loures-Ribeiro A (2013). Ethno-ornithology and conservation of wild birds in the semi-arid Caatinga of northeastern Brazil. J Ethnobiol Ethnomed.

[73] Teixeira PHR, Thel T, Ferreira J, Júnior S, Júnior W, Neves R (2014). Local knowledge and exploitation of the avian fauna by a rural community in the semi-arid zone of northeastern Brazil. J Ethnobiol Ethnomed.

[74] Farooq A (2012). AK Kayani. Prevalence of Superstitions and other Supernaturals in Rural Punjab: A Sociological Perspective. Res J South Asian Stud.

[75] Srithi K, Balslev H, Wangpakapattanawong P, Srisanga P, Trisonthi C (2009). Medicinal plant knowledge and its erosion among the Mien (Yao) in northern Thailand. J Ethnopharmacol.

[76] Bibi T, Ahmad M, Tareen RB, Tareen NM, Jabeen R, Rehman S-U, Sultana S, Zafar M, Yaseen G (2014). Ethnobotany of medicinal plants in district Mastung of Balochistan province-Pakistan. J Ethnopharmacol.

[77] Padmanabhan PSujana K (2008). Animal products in traditional medicine from Attappady hills of Western Ghats. Indian J Tradit Knowl.

[78] Lohani U (2010). Man-animal relationships in Central Nepal. J Ethnobiol Ethnomed.

[79] Alves RR, Rosa IL, Santana GG (2007). The role of animal-derived remedies as complementary medicine in Brazil. Bioscience.

[80] Benítez G (2011). Animals used for medicinal and magico-religious purposes in western Granada Province, Andalusia (Spain). J Ethnopharmacol.

[81] Alves RR, Neto NAL, Brooks SE, Albuquerque UP (2009). Commercialization of animal-derived remedies as complementary medicine in the semi-arid region of Northeastern Brazil. J Ethnopharmacol.

[82] Oliveira ES, Torres DF, Brooks SE, Alves RR (2010). The medicinal animal markets in the metropolitan region of Natal City, Northeastern Brazil. J Ethnopharmacol.

[83] Chakravorty J, Meyer-Rochow VB, Ghosh S (2011). Vertebrates used for medicinal purposes by members of the Nyishi and Galo tribes in Arunachal Pradesh (North-East India). J Ethnobiol Ethnomed.

[84] Betlu ALS (2013). Indigenous knowledge of zootherapeutic use among the Biate tribe of Dima Hasao District, Assam, Northeastern India. J Ethnobiol Ethnomed.

